# One size doesn’t fit all: Attitudes towards work modify the relation between parental leave length and postpartum depression

**DOI:** 10.1007/s00737-023-01374-5

**Published:** 2023-09-22

**Authors:** Christine Y. Chang, Sabrina R. Liu, Laura M. Glynn

**Affiliations:** 1 Department of Psychology, Chapman University, Orange, CA USA; 2 Department of Human Development, California State University, San Marcos, San Marcos, CA USA

**Keywords:** Parental leave, Pregnancy, Postpartum depression, Maternal health, Employment status

## Abstract

The present study aimed to investigate the relationship between parental leave length and maternal depressive symptoms at six- and twelve-months postpartum and whether this relation was influenced by women’s attitudes towards leave, whether leave was paid or unpaid, and the reason they returned to work. The sample included 115 working women recruited during pregnancy as part of a larger longitudinal study. Analyses revealed that maternal attitudes toward leave influenced the association between leave length and depressive symptoms. Specifically, longer leaves were associated with increased depressive symptoms for women who missed their previous activities at work. Furthermore, women who missed work and had leave for 16 weeks or more, exhibited higher depressive symptoms at six- and twelve-months. Last, results also indicated that women who returned to work solely for monetary reasons exhibited more depressive symptoms at six-months postpartum than those who returned to work for other reasons. This study is among the first to show that women’s attitudes towards parental leave and their individual reasons for returning to work are important factors to consider that may have potential implications for parental leave policies.

## Introduction

For many women, parenthood involves a series of transitions, including identity, biological, and occupational changes. These adjustments render the postpartum period a particularly sensitive time for emergence or exacerbation of mental health disorders (Glynn et al. 2018). The manner in which women navigate this transition and their postpartum mental health are further dependent on a variety of psychosocial factors such as familial and marital relationships (Qi et al. 2022; Clout and Brown 2016), social support (Qi et al. 2022; Gjerdingen and Chaloner 1994; Hetherington et al. 2018), breastfeeding status (Hahn-Holbrook et al. 2013; Watkins et al. 2011; Lara-Cinisomo et al. 2017), and socioeconomic status (Goyal et al. 2010). Determinants of postpartum depression are critical to investigate as postpartum depression is linked to a host of negative outcomes for mother and child, including adverse maternal physical health (Slomian et al. 2019), maternal suicidal ideation (Reid et al. 2022; Slomian et al. 2019), disrupted mother-child bonding (Kasamatsu et al. 2020; Edhborg et al. 2011), compromised infant and maternal health-related quality of life (Slomian et al. 2019; Darcy et al. 2011), poorer child cognitive outcomes (Urizar and Muñoz 2022; Piteo et al. 2012), insecure child attachment (Śliwerski et al. 2020), and delayed child growth (Farías-Antúnez et al. 2018).

### Parental leave and maternal mental health

For working parents, parental leave is a unique aspect of the postpartum experience that can affect mental health and the establishment of healthy parent-child attachment relationships (Clark et al. 1997, Plotka and Busch-Rossnagel 2018; Petts et al. 2020). In particular, shorter maternal leaves have previously been associated with increased maternal depressive symptoms (Chatterji and Markowitz 2012; Hewitt et al. 2017; Mandal 2018), anxiety (Hyde et al. 1995), psychological distress (Whitehouse et al. 2013), greater negative affect (Clark et al. 1997), and physical health challenges (Chatterji and Markowitz 2012, Hewitt et al. 2017). In addition to maternal health, shorter parental leaves can heighten risk for adverse child outcomes, such as increased infant mortality rates (Ruhm 2000; Nandi et al. 2016) and higher likelihood of child externalizing problems (Berger et al. 2005). Evidence has shown that whether leave is paid determines health impacts as well. Unpaid or shorter lengths of paid leave are correlated with increased maternal depression (Chatterji and Markowitz 2012), psychological distress (Whitehouse et al. 2013), compromised physical health (Chatterji and Markowitz 2012; Hewitt et al. 2017), and higher risk of being hospitalized postpartum (Jou et al. 2018). While there is research linking shorter and non-paid parental leaves with adverse outcomes, policies at the federal and international level vary greatly. The current standard of leave for the United States is based on the Family Medical Leave Act, which provides employees with twelve weeks of unpaid leave (Waldfogel 1999), while the current standard of leave for European countries based on the European Union is 16 paid weeks (Your Europe). Due to the lack of standard paid leave in the United States many women are forced to return to work for monetary reasons rather than when they feel ready to return (Han and Waldfogel 2003).

Although there are clear benefits of parental leave for both maternal and child health and well-being, not all research to date has shown a positive relationship between parental leave length and maternal mental health outcomes. For example, Dagher and colleagues (2014) documented a U-shaped relationship between leave and depressive symptoms – shorter leaves and leaves over 180 days were related to increased postpartum depressive symptoms. Klein and colleagues (1998), measured women’s attitudes towards their work and family roles prior to giving birth, finding that among women who valued their success in work more than their success in their family, longer leaves were predictive of higher levels of depressive symptoms. Individual risk factors such as marital concerns and unrewarding jobs have also been linked with higher levels of depression when in conjunction with short leaves (Hyde et al. 1995). Further research has shown that greater work flexibility can protect against a negative relationship between leave length and postpartum mental health (Newkirk et al. 2020). In sum, the association between leave and mental health may be dependent on a variety of individual difference factors including preferences, values, and familial and occupational responsibilities.

### Theoretical background

The mixed findings in the existing literature suggest that the assumption that longer leaves are better for maternal mental health does not universally apply to all women and this relation is likely more nuanced. From a theoretical perspective, there are two opposing arguments that may be applicable. The scarcity hypothesis suggests that humans have a predetermined amount of time and energy and each role that an individual has depletes their fixed amount of time and energy, leading to adverse psychological outcomes (Goode 1974; Froberg et al. 1986; Elgar and Chester 2007). Considered in the context of motherhood, a woman who adds the parental role to their employment role may be more susceptible to depression. A contrasting theory, the enhancement hypothesis, suggests that rather than leading to a depletion in energy, additional roles can complement existing roles by providing more pathways for empowerment, identity, support, and morale; each of which are associated with more optimal mental health (Baruch et al. 1987; Pao et al. 2019; Elgar and Chester 2007). Thus, the addition of the role of parent to a working individuals existing suite of roles would be expected to buffer or protect from postpartum depression.

### Current study

Building upon the enhancement theory and the view that additional roles could be beneficial, women who find fulfillment through their roles outside of the home may be more motivated to return to work early. While there is evidence to show that short parental leaves increase susceptibility to depression, research has also established that working mothers have more positive mental and physical health compared to non-working mothers (Rout et al. 1997; Buehler and O'Brien 2011; Frech and Damaske 2012). Beyond the understanding that very short and unpaid leaves are detrimental for maternal mental health, gaps remain in the literature regarding at which point in the postpartum period returning to work may exert salutary effects on mental health, and whether this timeframe is dependent on individual difference factors such as maternal attitudes towards leave and work. Previous studies have largely failed to acknowledge the variety of attitudes women have towards their work and leave and the reasons why they do or do not return.

Therefore, the present study aimed to investigate: 1) the association of parental leave length, maternal attitudes towards leave, paid vs. unpaid leave, and reason for returning to work (monetary vs other) with maternal mental health; 2) whether depressive symptoms differed between women who did and did not receive 12 or 16 weeks of leave (two common leave lengths); and 3) whether the relation between leave length or minimum leave length and mental health was influenced by mothers’ attitudes towards their leave, the reason they returned to work, or whether their leave was paid or unpaid.

The hypotheses for this study were as follows: (1) Women who returned to work solely for monetary reasons and women who received no paid leave would exhibit higher depressive symptoms compared to women who returned for other reasons and received paid leave. (2) There would be a negative relationship between leave length and maternal postpartum depressive symptoms, with longer parental leaves being associated with decreased postpartum depressive symptoms. (3) The negative relation between parental leave length and postpartum depressive symptoms would be less robust for women who felt confined or isolated at home while on leave, missed their previous activities at work, returned to work for reasons that were not monetary, and received paid leave.

## Method

### Participants and procedure

**Fig. 1:**
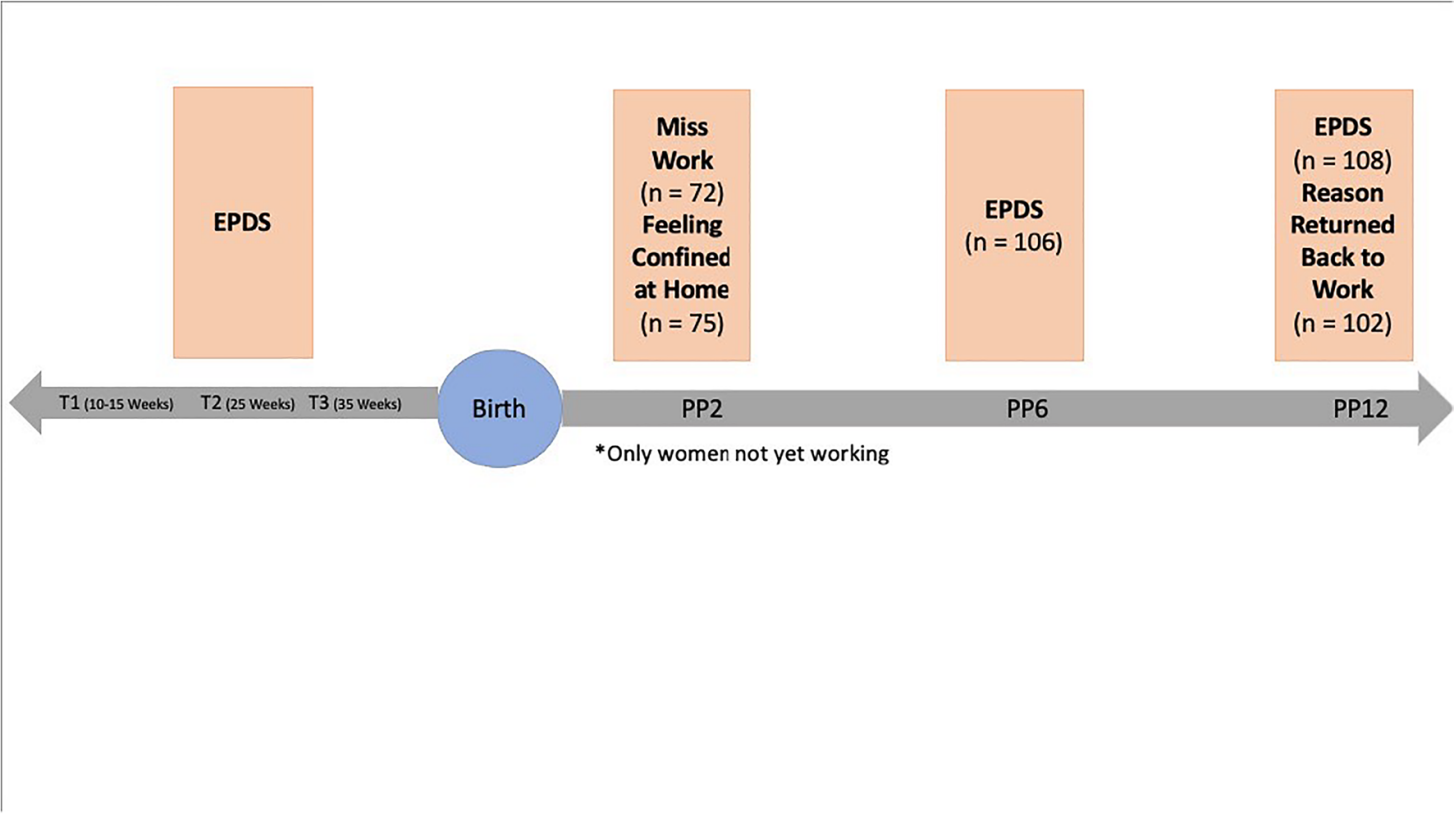
Flowchart illustrating longitudinal data collection. *Note*. **Abbreviations: T1:** Time 1. **T2:** Time 2. **T3:** Time 3. **PP2:** Two-months postpartum. **PP6:** Six-months postpartum. **PP12:** Twelve-months postpartum. **EPDS**: Edinburgh Postnatal Depression Scale. **Miss work**: Please tell me how true the following statement is for you: You miss working and your previous activities at work. **Feeling confined at home**: Please tell me how true the following statement is for you: You feel confined or trapped staying at home. **Reason returned back to work:** Some women return to work because they need money, but others return to work because they want to, or for other reasons. Why did you decide to go back to work?

This study utilized data from an ongoing longitudinal study of prenatal and early life experience and child development. Participants were recruited from large medical centers in Southern California during their first trimester of pregnancy. At initial recruitment, inclusion criteria were: (a) adult (≥ 18 years of age), (b) English-speaking, (c) intrauterine, singleton pregnancy, (d) absence of uterine or cervical abnormalities, (e) absence of conditions such as endocrine, hepatic, or renal disorders, or use of corticosteroid medication, and (f) absence of self-reported abuse of tobacco, alcohol, or recreational drugs in the pregnancy. For this study, an additional inclusion criterion comprised women who were working during their pregnancy, resulting in a final sample of 115 women. At initial recruitment in pregnancy, depression levels in women who were working (*M* = 5.75, *SD* = 4.82) versus those who were not working during pregnancy (*M* = 6.48, *SD* = 4.57) did not differ (t(217) = -1.09, *p* = 0.276). Data were collected in person throughout the years of 2014 to 2018. See Fig. 1 for a flowchart summarizing data collection. All study procedures were reviewed and approved by the University’s Institutional Review Board. Women in this study were asked questions about their attitudes towards their leave at two- months postpartum and their depressive symptoms were assessed at six- and twelve- months postpartum.

### Measures

#### Leave characteristics: length and pay

At postpartum visits, women were queried about their work status and assigned a leave length in weeks postpartum. For analyses examining depressive symptoms at six-months post-partum, participants who had a leave length longer than 24 weeks (e.g. six- months) were assigned 24 weeks as their leave length. For participants who had not returned to work postpartum by twelve months, their leave was coded as 52 weeks when examining symptoms at twelve- months postpartum.

Women who had returned back to work by twelve- months postpartum (*n* = 102) were asked the following question about why they returned back to work: “Some women return to work because they need money, but others return to work because they want to, or for other reasons. Why did you decide to go back to work?” Responses were coded as “Monetary only” and “Other.”

#### Occupational prestige

Occupational prestige was coded using an index developed out of the National Opinion Research Center (NORC; Nakao and Treas 1994). Scores range from 0–100 with higher scores indicating more prestige. The distribution of occupational prestige is shown in Table 1.

**Table 1:** Sociodemographic and Employment Characteristics

	Mean (SD), Median; Quartile Deviation, or % (n)
Age	29.27 (5.48)
Education	
*High School or Less*	14.8% (*n* = 17)
*Some College*	19.1% (*n* = 22)
*Associate’s or Vocational Degree*	27.0% (*n* = 31)
*4-Year College Degree*	17.4% (*n* = 20)
*Graduate Degree*	21.7% (*n* = 25)
Race/Ethnicity	
*African-American or Black*	3.5% (*n* = 4)
*Asian*	10.4% (*n* = 12)
*Hispanic or Latina*	43.5% (*n* = 50)
*Non-Hispanic White*	33.9% (*n* = 39)
*Multi-Ethnic*	8.7% (*n* = 10)
Income to Needs Ratio	337.50; 227.63
Parity	
*Primiparous*	54.8% (*n* = 63)
*Multiparous*	45.2% (*n* = 52)
Cohabitation status	
*Cohabitating with child’s father*	86.1% (*n* = 99)
*Not cohabitating with child’s father*	13.9% (*n* = 16)
Occupational Prestige*	
*20–35*	20.4% (*n* = 21)
*35–50*	39.8% (*n* = 41)
*50–65*	24.3% (*n* = 25)
*65–80*	12.6% (*n* = 13)
*80–95*	2.9% (*n* = 3)
Length of Parental Leave	14.00; 8.00
Paid vs. Unpaid Leave Status	
*Paid Leave*	66.1% (*n* = 76)
*Unpaid Leave*	28.7% (*n* = 33)
12 Weeks of Leave	
< *12 Weeks*	32.2% (*n* = 37)
> */* = *12 Weeks*	67.8% (*n* = 78)
16 Weeks of Leave	
< *16 Weeks*	50.4% (*n* = 58)
> */* = *16 Weeks*	49.6% (*n* = 57)
Feeling Confined at Home	1.95 (0.99)
Miss Work	2.51 (0.93)
Reason Women Returned to Work	
*Money*	50% (*n* = 51)
*Other*	50% (*n* = 51)
Depressive Symptoms – 6 Months	6.37 (4.64)
Depressive Symptoms – 12 Months	6.77 (4.97)

Occupational prestige was coded using an index developed out of the National Opinion Research Center (NORC; Nakao and Treas 1994). Scores range from 0–100 with higher scores indicating more prestige. Examples of representation in these categories are: 28.08 = Waiters and Waitresses; 32.33 = Crossing Guards; 45.73 = Teachers; 59.99 = Police; 66.48 = Registered Nurses; 86.05 = Physicians

#### Attitudes towards leave

Among the 75 participants who had not returned to work at two- months postpartum they were interviewed and asked the following questions at two-months postpartum: “Please tell me how true the following statement is for you: ‘You feel confined or trapped staying at home.’” and “Please tell me how true the following statement is for you: ‘You miss working and your previous activities at work.’” Participants responded to each question on a five-item Likert scale from one, “Not at all true” to five, “Completely true.”

#### Depressive symptoms

Maternal depressive symptoms were measured at six- and twelve- months postpartum using the Edinburgh Postnatal Depression Scale (EPDS). The EPDS is a ten-item scale that has been used widely to assess postpartum depression and has been validated in multiple studies with different populations (Hahn-Holbrook et al. 2018). The EPDS displayed strong internal consistency in this study with the value of the Cronbach’s Alpha being α = 0.85 at six- months and α = 0.83 for twelve- months.

### Data analyses

The following variables were examined as potential covariates, given previous literature suggesting their relation to maternal depressive symptoms postpartum: cohabitation status with infant’s father (Kiernan and Pickett 2006), occupational prestige (Segre et al. 2007), parity (Martínez-Galiano et al. 2019), age (Bottino et al. 2012) and income to needs ratio (Beck 2001) (Van Niel et al. 2020). Income to needs ratio is calculated from total family income in the last year divided by the federal guidelines for poverty, based on individual household size. None of the potential covariates reached threshold (*p* < 0.05) for inclusion in subsequent analyses testing study hypotheses (see Table 2).

**Table 2:** Bivariate Correlations of Leave Characteristics, Demographic Information, and Depressive Symptoms

	1	2	3	4	5	6	7	8	9	10	11	12	13	14
1. Leave Length	1.00													
2. 12 Weeks of Leave	0.58**	1.00												
3. 16 Weeks of Leave	0.69**	0.68**	1.00											
4. Paid vs. Unpaid Leave	-0.34**	-0.01	-0.22*	1.00										
5. Feeling Confined at Home	-0.04	-0.02	-0.07	-0.03	1.00									
6. Miss Work	0.05	0.00	-0.01	-0.16	0.44**	1.00								
7. Reason Women Returned to Work	0.04	-0.02	-0.06	0.00	0.04	-0.03	1.00							
8. Occupational Prestige	-0.07	0.10	-0.04	0.22*	0.02	-0.03	-0.16	1.00						
9. Maternal Age	-0.10	-0.01	-0.10	0.05	-0.16	0.08	0.02	0.48**	1.00					
10. Parity	-0.17	0.01	-0.05	0.32**	-0.03	-0.05	-0.19	0.02	0.25**	1.00				
11. Income to Needs Ratio	0.04	0.10	0.10	0.12	0.18	-0.02	-0.06	0.57**	0.45**	-0.11	1.00			
12. Cohabitation Status	-0.03	-0.06	-0.04	-0.10	0.10	0.07	0.34**	0.02*	0.29**	0.11	0.18	1.00		
13. Depressive Symptoms – 6 Months	0.10	-0.08	0.03	-0.15	0.21	0.15	0.10	0.05	0.03	0.09	-0.14	-0.05	1.00	
14. Depressive Symptoms – 12 Months	-0.07	0.10	0.04	0.22*	0.02	-0.03	-0.16	0.01	0.08	0.08	-0.07	0.12	0.64**	1.00

*p ≤ 0.05, **p ≤ 0.01

Pearson bivariate correlations using IBM SPSS v. 27 were used for analysis

Prior to analysis, tests for data normality were conducted to confirm depression at six-months and twelve-months appeared to be normally distributed with the skewedness test statistic being 0.64 for six-months and 0.66 for twelve-months. Pearson’s bivariate correlations were conducted to assess whether leave length, paid vs. unpaid leave, reason women returned to work, feeling confined at home, or feelings of missing work were associated with maternal depressive symptoms at six – and twelve – months postpartum. To assess whether feelings of being confined at home, missing work, paid vs. unpaid leave, or reason women returned to work moderated the relation between leave length or a minimum leave length and maternal depressive symptoms postpartum, an interaction effect was modeled using linear regressions (forced entry method). Significant interactions were visualized by plotting postpartum maternal depressive symptoms at two values of the predictor and moderator, representing high and low. For binary variables, the only two possible values were utilized. For continuous variables, values were assigned by taking one standard deviation above and below the mean (Hayes 2018).

We also examined whether there were differential effects based on receipt of a minimum leave length of 12 weeks (the leave length guaranteed by the United States’ Family Medical and Leave Act; Waldfogel 1999) or 16 weeks (the European Union’s standard guaranteed leave length; Your Europe).

All data were analyzed through IBM SPSS v27 and any data exclusions, sample sizes, manipulations, and measures have been reported. This study’s design and analysis were not pre-registered and any inquiries regarding data access, analysis code, and research materials should be made by emailing the corresponding author.

## Results

### Sample characteristics

Participant demographic information and sample characteristics is presented in Table 1.

### Examination of main effects

Results revealed no statistically significant bivariate correlations between leave length, minimum leave length, feeling confined or trapped at home, missing work, or paid vs. unpaid leave and maternal depressive symptoms at six- and twelve- months postpartum (See Table 2). However, there was an association between the reason women went back to work and depressive symptoms at six-months postpartum, with women who returned to work for monetary reasons displaying higher depressive symptoms (r = 0.34, *p* < 0.001). Even when accounting for income to needs ratio, this association remained statistically significant (partial r = 0.32, *p* = 0.002).

### Moderation models

**Fig. 2:**
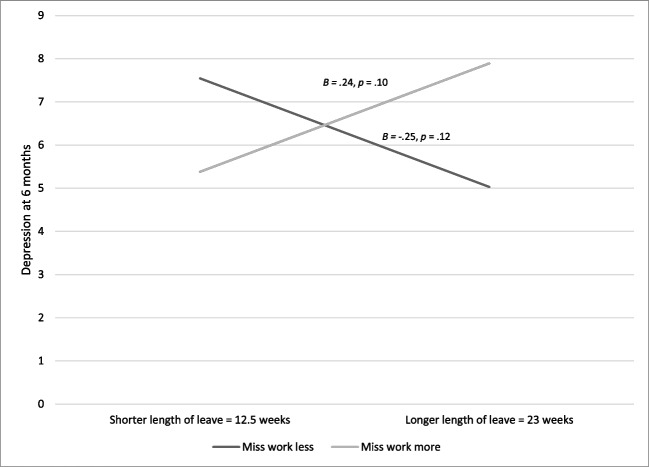
Missing Work Modifies the Association between Leave Length and Postpartum Depressive Symptoms. *Note. n* = 70. Mean leave length: 17.75, *SD*: 5.25. Mean miss work score: 2.5, *SD*: 0.92 In the linear regressions, and the simple slopes analyses shown here, both leave length and missing work were included as continuous variables. In order to visualize the data, each was plotted at one standard deviation above and standard deviation below the mean (Leave length = 12.5 weeks versus 23 weeks. Miss work = 1.58 versus 3.42)

**Fig. 3:**
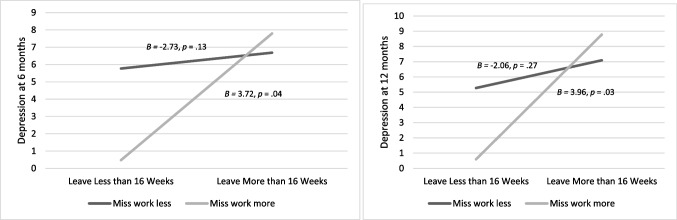
Missing Work Modifies the Association between Leave Length (16 weeks) and Postpartum Depressive Symptoms at 6 and 12 months. *Note.* Postpartum depressive symptoms at 6 months: *n* = 70. Postpartum Depressive Symptoms at 12 months: *n* = 68. Mean miss work score: 2.5, SD: 0.92. To visualize the data, miss work was plotted one standard deviation above the mean (3.42) and one standard deviation below the mean (1.58)

Missing work moderated the relation between leave length and maternal depressive symptoms at six- months postpartum (length X missing work *t* = 2.35, *p* = 0.022; See Table 3). For women who missed their previous activities at work, longer leaves were associated with increased depressive symptoms at six- months postpartum (see Fig. 2). Additionally, among women who missed work, when they had 16 weeks of leave or more, they exhibited higher depressive symptoms at six- months (16 weeks X missing work *t* = 2.56*, p* = 0.013*;* See Table 4) and twelve- months postpartum (16 weeks X missing work *t* = 2.33*, p* = 0.023; See Table 4) compared to women who received less than 16 weeks. Among women who missed work less, leaves shorter than 16 weeks were associated with higher levels of depressive symptoms (See Fig. 3). In contrast, when women received leave lengths of 12 weeks of less, the extent to which women missed work did not have any association with depressive symptoms at six- or twelve- months postpartum. Feeling confined at home, paid vs. unpaid leave, and reason women went back to work did not moderate the relation between leave length and maternal depressive symptoms at either six- or twelve- months postpartum.

**Table 3:** Linear Regression Testing the Association Between Leave Length and Feelings Towards Leave on Depressive Symptoms

	Six- Months Postpartum				Twelve- Months Postpartum			
	Estimate	*SE*	Standardized Estimate	*P*	Estimate	*SE*	Standardized Estimate	*P*
Step 1 (6 months): *F*(3,66) = 1.97, *p* = 0.127, *R*^*2*^ = 0.08 (12 months): *F*(3,64) = 1.54, *p* = 0.213, *R*^*2*^ = 0.07								
Constant	6.51	0.58		0.000	6.71	0.58		0.000
Missing Work	0.18	0.63	0.03	0.776	0.69	0.65	0.13	0.297
Leave Length	-0.00	0.11	-0.00	0.994	0.03	0.04	0.10	0.426
Leave Length X Missing Work	0.26	0.11	0.28	0.022*	0.07	0.05	0.20	0.107

PP6: *n* = 70. PP12: *n* = 68. Linear regressions using forced entry in IBM SPSS v. 27 were used for analysis

**Table 4:** Linear Regression Testing the Association Between 16 Weeks of Leave and Feelings Towards Leave on Depressive Symptoms

	Six- Months Postpartum				Twelve- Months Postpartum			
	Estimate	*SE*	Standardized Estimate	*P*	Estimate	*SE*	Standardized Estimate	*P*
Step 1 (6 months): *F*(3,66) = 2.35, *p* = 0.080, *R*^*2*^ = 0.10 (12 months): *F*(3,64) = 2.50, *p* = 0.067, *R*^*2*^ = 0.11								
Constant	6.23	1.02		0.000	6.15	1.02		0.000
16 Weeks	0.50	1.23	0.05	0.685	0.95	1.24	0.09	0.446
Missing Work	-2.09	1.14	-0.40	0.073	-1.63	1.21	-0.30	0.183
16 Weeks X Missing Work	3.48	1.36	0.55	0.013*	3.32	1.43	0.52	0.023*

PP6: *n* = 70. PP12: *n* = 68. Linear regressions using forced entry in IBM SPSS v. 27 were used for analysis

## Discussion

Expanding on previous research examining the relationship between parental leave length and maternal mental health postpartum, this study determined how a woman’s attitudes towards leave and reasons for returning to work might affect the relationship between parental leave length and postpartum depressive symptoms. Findings indicated that the reason women went back to work played a significant role in postpartum mental health. Specifically, returning to work solely for monetary reasons was associated with higher depressive symptoms at six-months, even after consideration of household income. This association was not present with depressive symptoms at 12-months postpartum and future research will be required to confirm whether a true timing effect exists, indicating that the negative mental health impacts of returning to work for monetary reasons dissipate as the first postpartum year progresses. Analyses further revealed that the relationship between leave length and depressive symptoms was dependent on a woman’s attitudes towards their leave. For women who missed their previous activities at work, longer leaves were associated with increased depressive symptoms. The opposite was true for women who did not miss their previous activities at work; longer leaves were associated with decreased depressive symptoms. Our study also found that women who had at least 16 weeks of leave and missed work, exhibited higher depressive symptoms at six- and twelve- months postpartum, indicating that when leave lengths extend beyond three- months, individual differences began to emerge. After this point, returning to work may be beneficial for a subgroup of women who desire to go back. In line with the enhancement hypothesis (Baruch et al. 1987; Pao et al. 2019), this present study presents evidence that returning to one’s working role can have a positive effect on women’s mental health postpartum.

By exploring novel questions about how a woman’s attitudes towards returning to work and her leave impacts postpartum depressive symptoms, these results extend current understandings of what specific aspects of parental leave have implications for maternal mental health. While this study is the first to our knowledge to operationalize a woman’s attitudes towards her leave and returning to work, existing literature has suggested that how a woman feels about her work may be an important aspect of parental leave. For example, Klein and colleagues (1998) documented that for women who valued success in their work relative to their family, longer leaves were associated with increased depressive symptoms. Our research builds on these results by directly asking women how they feel in the absence of their work during their parental leave as well as about their motivations for returning.

### Limitations

There are several limitations to consider in this study. First, this study had a relatively small sample size of just over 100 women. Perhaps a more important point to consider is that the women in this sample had relatively long parental leaves, with the median leave being 14 weeks. Further studies should examine these relations in larger populations with more diverse ranges of leave length. Another limitation of this study was that a woman’s attitudes towards leave was assessed using 1-item measures and a more comprehensive assessment could be beneficial in expanding understanding of the role of attitudes towards work and leave length in parental well-being. Furthermore, there are additional variables that could be important to consider in this context such as partner support, support in infant care, and flexible work policies. Finally, future studies may benefit from more directly testing the enhancement hypothesis, for example by measuring constructs related to empowerment, social support and identity.

### Implications

Our findings specifically demonstrate that a woman’s experiences of parental leave are related to her postpartum mental health. Although in this population leave length did not exhibit a direct effect with mental health, a woman’s attitudes towards her leave in conjunction with her leave length did predict her postpartum depressive symptoms. If women are missing their previous activities at work or have to go back to work for monetary reasons, they may be at a greater risk for compromised mental health. Recognizing the importance of these attitudes is critical, as poor postpartum mental health is not only related to poorer maternal physical health (Da Costa et al. 2006) and maternal suicidal ideation (Pope et al. 2013), but also has links to disrupted infant development (e.g., attachment styles, cognitive abilities, and mother-child interactions (Śliwerski et al. 2020; Urizar and Muñoz 2022; Kasamatsu et al. 2020).

In light of current parental leave policies, this study has important implications. First, it is notable that the United States is the only country in the Organization for Economic Co-operation and Development (OECD) to lack a comprehensive policy regarding paid maternity leave (Nandi et al. 2018). The United States does have the Family and Medical Leave (FMLA) policy which provides certain employees with 12 weeks of unpaid leave. However, this policy is heavily restricted, only being eligible for employees who work for an employer with 50 or more employees and worked for a minimum of 1,250 hours in the past year (Waldfogel 1999). Additionally, this policy only pays a percentage of an employee’s full compensation, and many employees may be unable to take 12 weeks of unpaid leave due to economic reasons or fears of termination. The current policy also does not guarantee that an individual will be restored to the same job they had prior to their leave (U.S. Department of Labor 2012). More recently, the House of Representatives passed the Build Back Better Act, guaranteeing four weeks of paid leave to all U.S. employees (117th Congress 2021). However, at the time of writing this manuscript, the bill is federally instilled but yet to be federally implemented. As the United States lacks a comprehensive parental leave policy, it is important to fill gaps on parental leave research to help guide and shape policy regarding parental leave.

Findings from our study and others suggest that leave policies can be improved to support postpartum mental health. Several leading organizations have begun to develop parental leave guidelines for mothers. For example, the American College of Obstetricians and Gynecologists recommends paid parental leave for at least six weeks (American College of Obstetricians and Gynecologists 2020), the International Labour Organization recommends a minimum of 14 paid and job-protected weeks (International Labour Organization), and the American Academy of Pediatrics endorses a six-to-nine-month minimum parental leave (NPR 2016). Our results illustrate that a woman’s experiences during leave are dependent on several factors and the best parental leave length is one that is individualized to fit a given woman’s needs. For example, parental leave policies that allow leave to be distributed over the course of a year at a mother’s discretion may be beneficial. Our findings suggest that a minimum of twelve weeks of leave can be beneficial for women, regardless of how they feel about their work. Only with longer leave lengths do differences in depressive symptoms emerge between women who missed and did not miss their previous activities at work. Last, our findings emphasize the need for paid parental leave as women can go back to work for a variety of reasons but going back to work solely for monetary reasons is negatively associated with mental health.

It is evident that parental leave is a crucial factor to consider for the well-being of working mothers postpartum, as well as the health and development of their children. More research focused on the individual differences that affect the benefits of leave is needed to provide support for the premise that parental leave is likely not a “one-size fits all” proposition and to inform the development of flexible leave policies and best practice guidelines. Ultimately, parental leave policies should be optimized to produce more beneficial outcomes for mothers, which in turn will have positive implications for children, families, and society as a whole.

## Data Availability

This study's design and and analysis were not pre-registered and any inquiries regarding data access, analysis code, and research materials should be made by emailing the corresponding author.
